# LibKey Nomad

**DOI:** 10.5195/jmla.2020.1017

**Published:** 2020-10-01

**Authors:** Matthew B. Hoy

**Affiliations:** 1 Hoy.Matt@mayo.edu, Associate Director, Mayo Clinic Libraries, Mayo Clinic, Rochester, MN

## Abstract

**LibKey Nomad.** Third Iron, P.O. Box 270400, St. Paul, MN 55127; https://thirdiron.com/libkey-nomad/; pricing: extension is free for users; institutional subscription to Third Iron products required; requirements: Google Chrome or Microsoft Edge browser (Firefox version currently under development).

## INTRODUCTION

Medical librarians spend a lot of time helping patrons locate portable document format files (PDFs) of journal articles. Library websites, link-resolvers, and full-text databases have been the tools of choice for this task for many years. These work well, but only if patrons begin their searches from their libraries. When patrons simply find a journal article via a web search or are sent a link, they have no quick and easy way of accessing their library's subscriptions. LibKey Nomad is a browser extension that can help solve this problem. This review describes what Nomad is, how it works, and what librarians need to do to make it available to their patrons. A short list of similar products is also included.

## WHAT IS LIBKEY NOMAD?

LibKey Nomad is a browser extension that quickly finds full-text copies of journal article PDFs as users browse the Internet. Nomad scans the content of pages that match its extensive list of publisher and scholarly database domains as they are loaded in users' browsers. If the page contains a journal article digital object identifier (DOI), Nomad will attempt to locate a PDF of the article. It looks for access in several places, including users' affiliated publisher holdings, aggregator databases, and open access repositories. Nomad overlays an icon in the lower left corner of users' browser screens that varies depending on whether or not a PDF is located:

- For articles with a direct path to the PDF file, Nomad displays a “Download PDF” button, which takes users to the file with one click.
- For articles where hypertext markup language (HTML) full text or a non-direct link to a PDF file is available, Nomad displays a “Link to Article” button.
- For articles that are located in repositories and are not the final versions but are accepted manuscripts, Nomad labels these as “Accepted Manuscripts” and indicates if it is a PDF or an article link as appropriate.
- When Nomad cannot locate full text for the article, it can optionally display an “Access Options” button that routes users to their libraries' link-resolvers to enable interlibrary loan.

All of these buttons can be branded with the user's home library name to make clear that the library is providing access.

**Figure 1:**
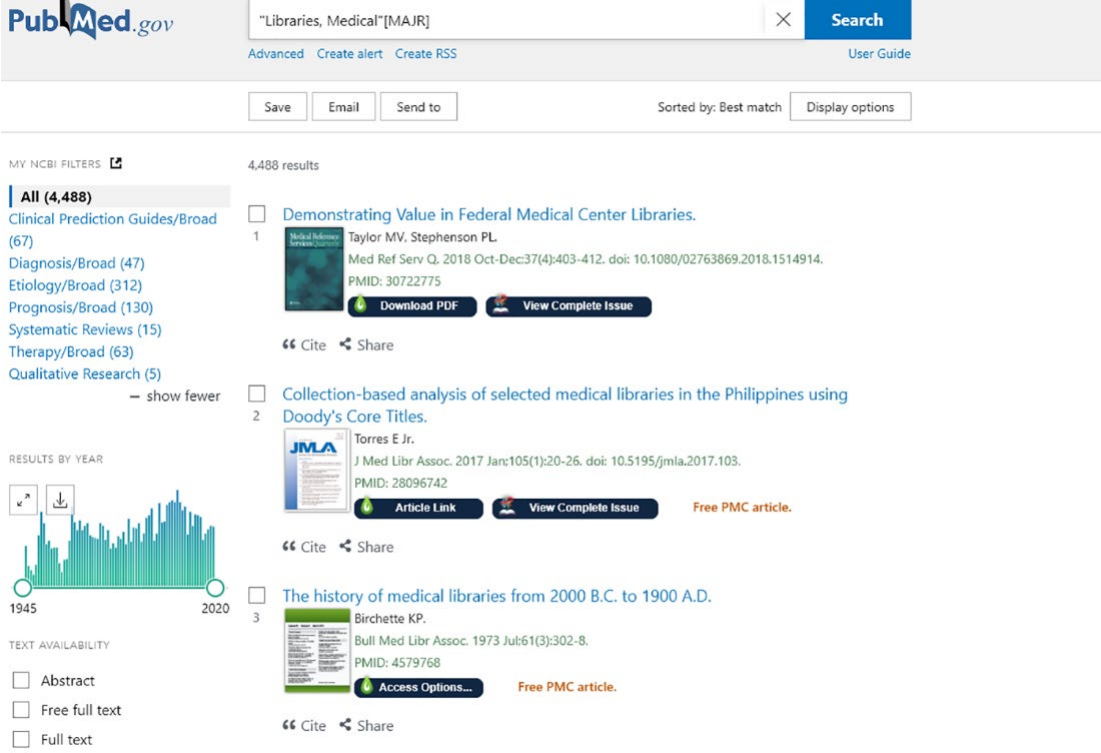
LibKey Nomad integration in PubMed

Nomad also has more direct integrations into several databases. When users with Nomad installed in their browsers conduct a search in PubMed, Wikipedia, Scopus, or Web of Science, the search results list is augmented with journal cover images and buttons for article access. There is also a button to open the complete issue of the corresponding journal in BrowZine, Third Iron's electronic journal reading product, with the article highlighted in its originally published context. A review of BrowZine appears in the October 2017 issue of the *Journal of the Medical Library Association (JMLA).* This button is only displayed if the journal is available in BrowZine. The journal cover images also link directly to the most recent issue of that journal if it is available in BrowZine (Figure 1).

## SETUP

LibKey Nomad requires very little configuration. Users can download the extension from the Chrome web store (quick link: libkeynomad.com). After downloading Nomad, they will be prompted to choose their home institutions. No other setup is required, and no user accounts, email addresses, or other personally identifiable information is collected or stored. Nomad makes user privacy one of its key selling points.

Librarians will need to ensure that they are currently subscribed to the Third Iron Complete product for their users to have access to LibKey Nomad. Proxy prefixes and holdings data will also need to be provided to Third Iron. Other forms of library authentication—including virtual private network (VPN), OpenAthens, and many other single sign-on systems—are also supported.

Nomad can also be installed by default for all users at an institution using Microsoft's Group Policy tools. This ensures that the tool is installed correctly and that users' home institutions are selected by default. Third Iron provides complete documentation for this setup process and can work with information technology (IT) departments to roll out Nomad quickly at an institutional level.

## SIMILAR PRODUCTS

There are several other browser extensions that serve a similar purpose to Nomad:

- Kopernio is a browser extension that functions similarly to Nomad by scanning for DOIs and adding download buttons to web pages. One key difference is that Kopernio does not have access to the holdings data of users' libraries. It simply tries to retrieve a copy of the PDF from known sources, some of which may work if users have access. Kopernio also offers user accounts and an online storage locker for PDFs. Kopernio was reviewed in the October 2019 *JMLA.*
- Unpaywall is an extension developed by Impactstory that finds open access versions of journal articles. Like Nomad, Unpaywall adds an icon to the screen indicating when a free, open-access PDF is available. Third Iron has partnered with Unpaywall to include links to open access articles in the Nomad extension. Unpaywall was reviewed in the April 2019 *JMLA.*
- Both the Google Scholar Button and the OA Button insert a button onto users' toolbars. When users find a web page containing a journal article DOI, they can click the toolbar button to locate copies of the article. Both tools search a number of sources for free and open access copies of journals articles, but neither has the ability to directly link to users' library entitlements. These tools also require users to intervene before displaying article availability.

## CONCLUSION

LibKey Nomad is an excellent tool for users who frequently search for PDFs. The integration of library holdings data makes Nomad fast and reliable, and the inclusion of the link-resolver button for items that are not available offers a seamless way to introduce interlibrary loan to users who might not normally use that service. Libraries that already subscribe to Third Iron's BrowZine product should definitely encourage their users to install Nomad, and those that do not subscribe may want to explore the possibility. Nomad makes finding PDFs painless and integrates into PubMed and other databases beautifully.

